# Enhanced Vitamin C Delivery: A Systematic Literature Review Assessing the Efficacy and Safety of Alternative Supplement Forms in Healthy Adults

**DOI:** 10.3390/nu17020279

**Published:** 2025-01-14

**Authors:** Philip C. Calder, Richard B. Kreider, Diane L. McKay

**Affiliations:** 1 School of Human Development and Health, Faculty of Medicine, University of Southampton, Southampton SO16 6YD, UK; 2 NIHR Southampton Biomedical Research Centre, University Hospital Southampton NHS Foundation Trust and University of Southampton, Southampton SO16 6YD, UK; 3 Exercise and Sport Nutrition Lab, Department of Kinesiology and Sports Management, Texas A&M University, College Station, TX 77843, USA; rbkreider@tamu.edu; 4 Friedman School of Nutrition Science and Policy, Tufts University, Boston, MA 02111, USA; diane.mckay@tufts.edu

**Keywords:** alternative supplement forms, Ester C, systematic literature review, vitamin C

## Abstract

Vitamin C is an antioxidant and is essential for immune function and infection resistance. Supplementation is necessary when a sufficient amount of vitamin C is not obtained through the diet. Alternative formulations of vitamin C may enhance its bioavailability and retention over traditional ascorbic acid. This systematic review consolidates the evidence on this and the effects on immunity and infection. A systematic literature search was conducted in October 2024 in Embase and Medline, focused on healthy adults (Population); oral forms of liposomal-encapsulated ascorbic acid, liposomal-encapsulated lipid metabolite ascorbic acid, calcium ascorbate, slow-release ascorbic acid, or lipid metabolite ascorbic acid (Intervention); compared to placebo/others (Comparison); in terms of bioavailability, absorption, vitamin C concentration in plasma, serum, and leukocytes, and impacts on tolerability, immunity, and infection (Outcome); and included randomized or non-randomized controlled trials, single-arm trials, and observational studies (Study design). Thirteen studies were included, several evaluating calcium ascorbate in combination with vitamin C metabolites, including L-threonate, referred to here as Calcium ascorbate EC (Ester C^®^; n = 7). No safety or tolerability concerns were noted with Calcium ascorbate EC vs. placebo or ascorbic acid. Calcium ascorbate EC showed better tolerability and fewer epigastric adverse events, improved quality of life, and induced favorable oxalate changes vs. ascorbic acid. Four studies reported leukocyte vitamin C concentration, some showing higher concentrations with Calcium ascorbate EC vs. ascorbic acid; seven reported more favorable plasma concentrations with the alternative forms over ascorbic acid or placebo; one reported higher serum vitamin C levels with vitamin C lipid metabolites than with Calcium ascorbate EC, calcium ascorbate, and ascorbic acid. No study reported retention in tissues. One study reported a favorable impact of Calcium ascorbate EC on immune parameters, and one found an association of Calcium ascorbate EC with fewer colds and a shorter duration of severe symptoms vs. placebo. Findings suggest that alternative vitamin C forms can improve leukocyte vitamin C, sometimes without affecting plasma levels. Most studies (77%) had a low risk of bias. In conclusion, the type and delivery modality of vitamin C can impact its bioavailability and functionality. Studies highlight the advantages of Calcium ascorbate EC over traditional ascorbic acid in terms of its tolerability and its potential to increase leukocyte vitamin C concentrations, crucial for immune function and protection against infection. However, further research is required to conclusively establish its effects on immune health.

## 1. Introduction

Vitamin C is crucial for immune system function [1,2,3,4] and protects against damage from reactive oxygen species [5,6]. Consequently, adequate cell and tissue stores of vitamin C maintain resistance to infections [5]. Decreased concentrations of vitamin C in immune cells, such as leukocytes, are associated with a diminished functional capacity of those cells [7].

Due to the inability of the human body to produce endogenous vitamin C, supplementation of the diet can be used to maintain adequate amounts of the nutrient [8]. Vitamin C supplements typically contain the ascorbic acid form, whose bioavailability is comparable with the form naturally occurring in foods [9,10,11]. However, this conventional form of vitamin C is susceptible to stomach acid, resulting in reduced bioavailability and shorter duration of retention in the blood and in immune cells, including leukocytes [12]. Its low chemical stability in the gastrointestinal tract necessitates an efficient delivery mode for vitamin C supplements [13]. Other forms of vitamin C supplements include sodium ascorbate, calcium ascorbate, other mineral ascorbates, ascorbic acid with bioflavonoids, and combination products [14].

Commercially available oral supplemental forms of vitamin C alternative to conventional ascorbic acid include Ester C^®^, which is a combination of calcium ascorbate and vitamin C metabolites, including L-threonate, and is termed as Calcium ascorbate EC in this publication. This form of vitamin C has a neutral pH (nonacidic) and has been shown in clinical studies to have a longer retention (up to 24 h) in immune cells (leukocytes) [15,16,17]. The other alternative forms include vitamin C lipid metabolites (PureWay C^®^), a composition that combines lipid metabolites (fatty acids) and citrus bioflavonoids with ascorbic acid for improved delivery efficiency to the human body [18]; liposomal-encapsulated L-ascorbic acid forms, encompassing different modern microencapsulation techniques that have been developed, involving the encapsulation of vitamin C with biopolymers and lipids like liposomes, which are expected to reduce the degradation of vitamin C, facilitate its controlled release, and enhance its absorption [19,20,21,22]; and sustained-release oral forms, utilizing a delivery format suited for sustained release over a 12 h period, such as the novel sustained-release L ascorbic acid (C-Fence^®^) [23].

About 70% of vitamin C in the blood is in plasma/serum and erythrocytes, which do not concentrate vitamin C from plasma, while 30% is in leukocytes, which have a marked ability to concentrate vitamin C from plasma [24]. Although vitamin C levels in plasma or serum are easily determined, they are not a true reflection of tissue content or leukocyte levels [17,24]. Plasma levels of vitamin C increase in the period after it is consumed and reflect the amount absorbed directly from the digestive tract, whereas the efficiency of vitamin C is determined by the level of its retention in cells and tissues, including leukocytes. The rapid uptake of vitamin C into plasma (or serum) and its retention in tissues and leukocytes are also key indicators of vitamin C supplementation efficacy and its potential for supporting the immune response. The storage of high levels of vitamin C in the body is important for the effective antioxidant properties it provides [24]. Leukocytes also maintain several times higher vitamin C concentrations than plasma and serum [24]. Various vitamin C forms are used in supplements (see earlier), with some claiming superiority over others in terms of bioavailability, absorption, plasma concentration, retention in the body and immune cells, and tolerability [15,18]. Despite these claims, there is a need to consolidate the available evidence to fully understand the potential benefits of each form.

The aim of this systematic literature review (SLR) is to consolidate the evidence around bioavailability, absorption, vitamin C concentration in plasma and leukocytes, and impacts on tolerability, immunity and infection in healthy adults with forms of vitamin C other than conventional ascorbic acid in order to determine if one form can be considered superior based on currently available research.

## 2. Materials and Methods

### 2.1. Study Design

This systematic review followed the Preferred Reporting Items for Systematic Reviews and Meta Analyses (PRISMA) guidelines. This review was not registered.

### 2.2. Research Question and Search Strategy

A systematic search was conducted in Embase and OVID Medline to identify published literature from 2000 up to October 2024. The research question was formulated using the following Population, Intervention, Comparison, Outcome, and Study design (PICOS) framework [25]: comparison of oral forms of liposomal-encapsulated ascorbic acid, liposomal-encapsulated lipid metabolite ascorbic acid, Calcium ascorbate EC, slow-release ascorbic acid, and lipid metabolite ascorbic acid (I); with placebo or any other intervention (C); in terms of bioavailability, absorption, raising plasma or serum vitamin C concentrations, retention in leukocytes, impact on immunity and infection, and tolerability (O); randomized controlled trial (RCT), non-RCT, single-arm study, and observational study (S); in healthy adults (≥18 years) (P). Keywords and free-text words used to search from OVID included vitamin C, ascorbic acid, Ester C, liposomal vitamin C, bioavailability, plasma concentration, absorption, tolerability, pharmacokinetics, RCT, clinical trial, observational study, and single-arm study. The detailed search string for Embase and OVID Medline is provided in the Supplemental Information (Table S1).

### 2.3. Eligibility Criteria

Articles that met the predefined inclusion/exclusion criteria (Table 1) were included.

### 2.4. Review Methodology

Titles and abstracts identified during the systemic search were reviewed by two independent reviewers against the pre-determined eligibility criteria. All publications with uncertainties were resolved either through reconciliation or arbitration by a third reviewer. Excluded publications were disregarded for data extraction and analysis of results. Full text versions of the shortlisted articles were retrieved and reviewed based on the same eligibility criteria for inclusion in the final report, while irrelevant records were excluded. The approach to resolve disagreements was the same as that during the abstract review process. All publications included after the full text review were retained for data extraction. The outcomes of the SLR were discussed.

### 2.5. Data Extraction

A standardized data extraction template was developed in Excel and the data extracted from the included studies were as follows: participant characteristics such as age, sex distribution, height, weight, body mass index (BMI), baseline plasma or serum vitamin C, and baseline leukocyte vitamin C. Effectiveness-related outcomes extracted were plasma or serum vitamin C levels, leukocyte vitamin C concentration, plasma C-reactive protein levels, plasma oxidized low-density lipoprotein, urine markers, impact on immunity or infection, pharmacokinetic parameters, and safety and tolerability.

### 2.6. Assessment of the Methodological Quality of the Studies

The quality of each study retained for data extraction was assessed to ensure that the conclusions and findings of this review are based on the best available evidence and that any potential sources of bias in the data are identified. The quality of the studies was assessed using the NICE checklist tool [26]. This tool is structured into four types of biases—selection bias, performance bias, attrition bias, and detection bias—and into key domains including randomization, treatment allocation, baseline characteristics, blinding, imbalances in dropouts, more outcome measures than reported, and intention-to-treat analysis.

## 3. Results

### 3.1. Search Results

**Figure 1:**
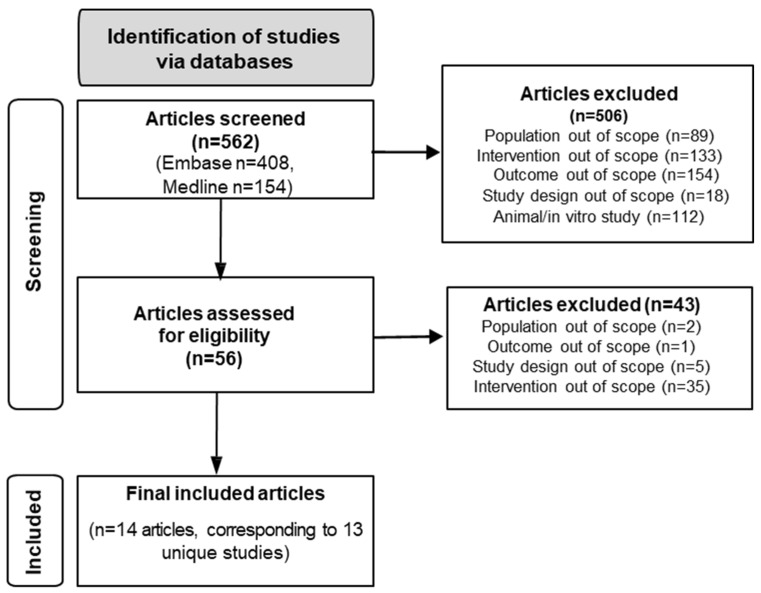
PRISMA flow diagram showing selection of articles for inclusion.

A total of 562 articles (published from 2000 up to October 2024) were identified via an electronic search using Embase and OVID Medline. Following title and abstract screening, 506 irrelevant articles were eliminated and 56 were reviewed for full text eligibility. Of these, 43 articles were excluded due to study design (n = 5), population (n = 2), intervention (n = 35), or outcomes (n = 1) not being relevant to SLR objectives. Finally, 14 articles (reporting on 13 primary RCTs) were deemed eligible for inclusion (Figure 1).

### 3.2. Characteristics of the Retrieved Studies: Population, Intervention, and Comparator

This review includes results from fourteen articles [12,16,27,28,29,30,31,32,33,34,35,36,37,38] reporting on a total of thirteen unique RCTs (two articles [16,38] report results from the same RCT) (Table 2). All studies included healthy populations; most included young or middle-aged adults and the mean age of the participants included in the different studies ranged from 24 to 48 years (Table 2). Two studies assessed only males [27,31]. Out of the thirteen unique studies, seven used Calcium ascorbate EC [27,28,30,32,33,34,38], one used lipid-metabolite ascorbic acid (PureWay C^®^) [35], two used sustained or slow-release ascorbic acid [29,31], and three used liposomal-encapsulated ascorbic acid [12,36,37] as an intervention. Studies compared Calcium ascorbate EC with ascorbic acid and placebo [27,28,30,32,33,34,38], and one study compared it with lipid metabolite, ascorbic acid, and calcium ascorbate [35]. Intervention studies on liposomal-encapsulated forms had non-liposomal vitamin C as the comparator [12,36,37]. Both studies with sustained-release vitamin C as the intervention were placebo-controlled [29,31]. Eight studies (nine articles) used single dosing [12,16,27,30,31,35,36,37,38], usually to follow pharmacokinetics [12,16,30,31,36,37]. Doses used were 250, 500, or 1000 mg. Five studies used chronic dosing over several days [28,29,32,33,34]; doses used were 1000 mg/day for 3 days [32], 1000 mg/day for 5 days and then 2000 mg/day for 5 days [33,34], 3000 mg/day for 14 days [29], and 500 mg/day for 60 days [28].

Participant characteristics and the baseline plasma, serum, or leukocyte vitamin C concentrations are shown in Table 2.

### 3.3. Characteristics of the Retrieved Studies: Outcomes

Table 3 describes the outcomes reported in each of the included studies. There was substantial variation in the reported metrics and outcomes across studies, with the most frequently reported outcomes being safety/tolerability and plasma vitamin C concentration.

**Table 2:** Baseline characteristics of the included studies.

Publication	Study Characteristics					Participant Characteristics		Baseline Vitamin C		
	Design	Intervention; Dose	Comparator; Dose	Cross-Over (Y/N)	Sample Size (n)	Age (y) Mean ± SD or Mean or Range	Sex Distribution (%Male/%Female)	Plasma Mean ± SD or Mean	Serum (mg/mL) Mean ± SD	Leukocyte Mean ± SD or Mean
Dickerson et al., 2024 [30]	RDB	CA EC; 250 mg single dose	AA; 250 mg single dose	Y	46	26 ± 10	41/59	CA EC: 3.2 ± 2.1 mg/mL AA: 3.1 ± 2.0 mg/mL	NR	CA EC: 7.5 ± 11.2 mg/mL AA: 6.1 ± 11.6 mg/mL
		CA EC; 500 mg single dose	AA; 500 mg single dose	Y	47	25 ± 9	49/51	CA EC: 4.9 ± 2.5 mg/mL AA: 5.4 ± 2.1 mg/mL	NR	CA EC: 2.0 ± 1.1 mg/mL AA: 3.1 ± 5.1 mg/mL
Pancorbo et al., 2008 [35]	RDB	Vitamin C lipid-metabolites (PureWay-C); 1000 mg single dose	CA EC; 1000 mg single dose AA; 1000 mg single dose CA; 1000 mg single dose	N	40 (10 per group)	21 to 50	NR	NR	Vitamin C lipid metabolites: 5.6 ± 0.9 CA EC: 6.0 ± 0.8 AA: 5.6 ± 0.6 CA: 5.0 ± 0.5	NR
Moyad et al., 2008 [27]	RDBPC	CA EC with 3% calcium threonate; 1000 mg single dose CA EC with 1% calcium threonate; 1000 mg single dose	Vitamin C: 1000 mg single dose Placebo	Y	15 (9 never smoked)	28	100/0	~60 mmol/L (never smoked) ~40 mmol/L (smokers)	NR	~80 mmol/10^8^ cells (never smoked) ~20–50 mmol/10^8^ cells (smokers)
Moyad et al., 2009 [33]	RDB	CA EC: 1000 mg/day for days 1–5, then 2000 mg/day for days 6–10	AA: 1000 mg/day for day 1–5 then 2000 mg/day for day 6–10	Y	50 (data reported for 34)	44	NR	CA EC: 48 ± 42 mmol/L AA: 42 ± 28 mmol/L	NR	NR
Rajat et al., 2022 [31]	RDBPC	AA sustained release; 500 mg single dose	Placebo	N	9	33 ± 7	100/0	NR	NR	NR
					9	25 ± 5	100.0	NR	NR	NR
Purpura et al., 2024 [37]	RDBPC	Liposomal vitamin C; 500 mg single dose	Placebo AA; 500 mg single dose	Y	27	36 ± 5	70/30	NR	NR	NR
Wen et al., 2022 [12]	RDB	Liposomal vitamin C (with lecithin); 1000 mg single dose	Liposomal vitamin C (with lecithin and high-pressure homogenization); 1000 mg single dose Vitamin C; 1000 mg single dose	Y	11	33 ± 8	45/55	Reported as 0 ± 0 mg/mL	NR	NR
Gopi and Balakrishnan 2021 [36]	ROL	Liposomal vitamin C; 1000 mg single dose	AA; 1000 mg single dose	Y	24	34 ± 7	NR	~10 mg/mL	NR	NR
Mitmesser et al., 2016 * [16]	RDBPC	CA EC: 1000 mg single dose	Placebo (PL) AA; 1000 mg single dose	Y: half followed EC -> AA -> PL and half followed AA-> PL -> EC	40 randomized; data provided for 30	18 to 60	53.3/46.7	EC -> AA -> PL: 6.9 ± 5.4 mg/mL AA -> PL -> EC: 4.7 ± 4.0 mg/mL	NR	EC -> AA -> PL: 9.3 ± 6.2 mg/10^8^ cells AA -> PL -> EC: 8.7 ± 4.1 mg/10^8^ cells
Mitmesser et al., 2014 * [38]	RDBPC	CA EC: 1000 mg single dose	Placebo AA; 1000 mg single dose	Y	36	18 to 60	50/50	NR	NR	NR
Ye et al., 2015 [34]	RDB	CA EC; 1000 mg/day for days 1–5, then 2000 mg/day for days 6–10	AA; 1000 mg/day for day 1–5 then 2000 mg/day for day 6–10	Y	50	NR	22/78	NR	NR	NR
Gruenwald et al., 2006 [32]	RDB	CA EC; 1000 mg/day for 3 days	AA; 1000 mg/day for 3 days	Y	50	46 ± 15	53/47	NR	NR	NR
Brody et al., 2002 [29]	RDBPC	AA sustained release; 3000 mg/day for 14 days	Placebo	N	AA: 54	26 ± 4	33.3/66.7	AA: 88 mmol/L	NR	NR
					Placebo: 54	24 ± 4	44.4/55.6	Placebo: 77.2 mmol/L	NR	NR
Van Straten and Josling 2002 [28]	RDBPC	CA EC; 500 mg/day for 60 days	Placebo	N	CA EC: 84	48	18/82	NR	NR	NR
					Placebo: 84	48	14/86	NR	NR	NR

Abbreviations: AA, ascorbic acid; CA, calcium ascorbate; CA EC, Calcium ascorbate EC; NR, not reported; PL, placebo; RDB, randomized double-blind; RDBPC, randomized double-blind placebo controlled; ROL, randomized open-label. Definitions of vitamin C alternate forms: Ester C^®^, Calcium ascorbate EC with vitamin C metabolites; PureWay C^®^, vitamin C lipid metabolites combining lipid metabolites (fatty acids) and citrus bioflavonoids with ascorbic acid. * Same study.

**Table 3:** Outcomes reported in the included studies.

Intervention	Publication	Plasma Vitamin C Concentration	Leukocyte Vitamin C Concentration	Serum Vitamin C Concentration	Pharmacokinetic Parameters	Impact on Immune Biomarkers	Safety and Tolerability
Calcium ascorbate EC (Ester C^®^)	Dickerson et al., 2024 [30]	Yes	Yes	No	Yes	Yes	Yes
	Mitmesser et al., 2016 * [16]	Yes	Yes	No	Yes	No	Yes
	Mitmesser et al., 2014 * [38]	Yes	Yes	No	No	No	No
	Ye et al., 2015 [34]	No	No	No	No	No	Yes
	Van Straten and Josling 2002 [28]	No	No	No	No	Yes (infection)	Yes
	Gruenwald et al., 2006 [32]	No	No	No	No	No	Yes
	Moyad et al., 2008 [27]	Yes	Yes	No	No	No	Yes
	Moyad et al., 2009 [33]	No	No	No	No	No	Yes
Vitamin C lipid metabolites (PureWay C^®^)	Pancorbo et al., 2008 [35]	No	No	Yes	No	No	Yes
Liposomal-encapsulated vitamin C	Purpura et al., 2024 [37]	Yes	Yes	No	Yes	No	Yes
	Wen et al., 2022 [12]	Yes	No	No	Yes	No	Yes
	Gopi and Balakrishnan 2021 [36]	Yes	No	No	Yes	No	Yes
Sustained-release vitamin C	Rajat et al., 2022 [31]	Yes	No	No	Yes	No	Yes
	Brody et al., 2002 [29]	No	No	No	No	No	Yes

* Different articles, reporting distinct outcomes but pertaining to the same primary study. Definitions of vitamin C alternate forms: Ester C^®^, Calcium ascorbate EC with vitamin C metabolites; PureWay C^®^, vitamin C lipid metabolites combining lipid metabolites (fatty acids) and citrus bioflavonoids with ascorbic acid.

Plasma vitamin C concentration was reported in seven studies (54%): one was concerned with sustained-release vitamin C [31], three with liposomal-encapsulated forms [12,36,37], and three (four articles) with Calcium ascorbate EC [16,27,30,38]. Four studies (31%) reported leukocyte vitamin C concentrations: three (in four articles) were concerned with Calcium ascorbate EC [16,27,30,38] and one with a liposomal-encapsulated form [37]. One study reported serum vitamin C concentration with lipid metabolite ascorbic acid [35]. Pharmacokinetic parameters were reported in six (46%) studies: one dealt with sustained-release vitamin C [31], three with liposomal-encapsulated forms [12,36,37], and two with Calcium ascorbate EC [16,30]. Pharmacokinetic parameters were captured as area under the curve (AUC 0–24 h), maximum observed concentration (Cmax), and time of maximum concentration (Tmax). One study investigated the effect of Calcium ascorbate EC on immune function by assessing neutrophil functionality (phagocytosis) and the number of different lymphocyte subsets and natural killer cells after ex vivo stimulation [30], while another captured the incidence and symptoms of the common cold [28]. All thirteen studies reported safety and tolerability data [12,16,27,28,29,30,31,32,33,34,35,36,37].

### 3.4. Summary of Evidence on the Efficacy of the Interventions

#### 3.4.1. Calcium Ascorbate EC (Ester-C^®^)

Moyad et al. [27] conducted a double-blind, placebo-controlled, four-way crossover design with 15 healthy males aged 18–39 years to compare the effects of different vitamin C formulations on plasma and leukocyte vitamin C levels. The study consisted of four separate phases, each lasting 24 h, with a 7-day washout period between phases. Participants received one of four 1000 mg oral preparations as a single dose: vitamin C alone, two separate formulations of calcium ascorbate with vitamin C metabolites (1% and 3% threonate), and placebo. Vitamin C concentrations in plasma and leukocytes were measured by high-performance liquid chromatography at baseline and at six sequential time periods over 24 h. All vitamin C interventions were significantly different from placebo for vitamin C in both plasma (*p* < 0.0001) and leukocytes (*p* < 0.0001), and there were few significant differences in plasma vitamin C levels across the preparations, regardless of the post-treatment time monitoring period. At 24 h, calcium ascorbate with metabolites significantly increased leukocyte vitamin C concentrations compared to ascorbic acid alone (1.3 to 1.7 times higher, *p* < 0.0001).

Mitmesser et al. [16] reported data from 30 participants (out of 40 enrolled) who received placebo, ascorbic acid (1000 mg), or Calcium ascorbate EC (1000 mg) in a double-blind, placebo-controlled crossover trial [16]. The study included three 24 h test periods, each anteceded by a 7-day washout period. Blood samples collected at baseline (0 h) and at 2, 4, 8, and 24 h post-dose were analyzed to assess the pharmacokinetics of plasma and leukocyte vitamin C. Calcium ascorbate EC resulted in higher plasma concentrations and a greater percent change from baseline compared to the placebo at all time points (*p* = 0.007 for percent change at 24 h, all others *p* < 0.001). No significant differences were found between Calcium ascorbate EC and ascorbic acid in plasma concentration; however, Calcium ascorbate EC resulted in a higher maximum plasma concentration (Cmax of 7.73 ± 3.12 µg/mL vs. 1.83 ± 2.07 µg/mL for placebo [*p* < 0.001] and 6.37 ± 2.26 µg/mL for ascorbic acid [*p* = 0.039]). Calcium ascorbate EC also showed a significant increase in leukocyte vitamin C concentration at 24 h post-dosing (*p* = 0.036), unlike ascorbic acid or placebo. During the investigational period, Calcium ascorbate EC led to a sustained retention of leukocyte vitamin C, with percent changes from baseline that were significantly higher at 8 and 24 h post-dosing compared to ascorbic acid and placebo.

As these previous studies had shown higher intracellular vitamin C in leukocytes 24 h after taking 1000 mg Calcium ascorbate EC compared to ascorbic acid [16,27], Dickerson et al. focused on comparing leukocyte vitamin C levels over 32 h in 93 participants after taking 250 mg (n = 46) or 500 mg doses (n = 47) from both sources [30]. Secondary goals included evaluating neutrophil function (phagocytosis) and immune cell numbers after ex vivo stimulation. The study found no significant differences in plasma and leukocyte vitamin C levels between ascorbic acid and Calcium ascorbate EC at 250 mg. However, at 500 mg, Calcium ascorbate EC significantly increased dehydroascorbic acid levels in plasma, indicating better absorption compared to ascorbic acid, suggesting that calcium ascorbate EC can enhance vitamin C bioavailability at higher doses. Also, at 500 mg, Calcium ascorbate EC improved neutrophil phagocytosis and increased natural killer cell counts, suggesting superior immune support over ascorbic acid. Pharmacokinetic analysis showed Calcium ascorbate EC had a greater volume of distribution and clearance from the blood compared to ascorbic acid, especially at a dosage of 500 mg, indicating its more effective utilization by the body. Overall, the study suggests that while lower doses of Calcium ascorbate EC and ascorbic acid are comparable, at higher doses, Calcium ascorbate EC may offer superior benefits in terms of immune function, with important implications for vitamin C supplementation in enhancing immune health and optimizing bioavailability [30].

Van Straten and Josling [28] studied 168 participants who were randomly assigned to receive either a placebo or a 1000 mg Calcium ascorbate EC supplement daily over a 60-day period. The average cold symptom length in days and the total number of colds per group were analyzed. Those in the Calcium ascorbate EC group had significantly fewer common cold episodes (37 in comparison with 50 in the placebo group, *p* < 0.05) and a shorter duration of severe symptoms compared to the placebo group (average of 1.8 vs. 3.1 days in the placebo group, *p* < 0.03). The lower infection risk reported with Calcium ascorbate EC in [28] is consistent with the reported improvements in immune cell function [30] and in leucocyte vitamin C reported in other studies [16,27].

#### 3.4.2. Vitamin C Lipid Metabolites (PureWay-C^®^)

Pancorbo et al. [35] conducted a prospective, randomized, double-blind trial with 40 participants randomly divided into equal treatment groups (n = 10) of four 1000 mg vitamin C formulations: ascorbic acid, calcium ascorbate, vitamin C lipid metabolites (PureWay-C^®^), and Calcium ascorbate EC [35]. Blood samples were collected at baseline and 1, 2, 4, 6, and 24 h post-dose. Vitamin C lipid metabolites led to the highest serum vitamin C levels, with significant increases at 1, 2, 4, and 6 h post-supplementation in comparison with calcium ascorbate. In contrast, Calcium ascorbate EC did not show a significant increase in serum vitamin C when compared to other vitamin C formulations, except at 1 and 4 h post-supplementation in comparison with calcium ascorbate. All formulations achieved peak absorption levels 2 h after supplementation, with only marginally higher levels 24 h later.

#### 3.4.3. Liposomal-Encapsulated Vitamin C

The three included studies reported efficacy data in terms of bioavailability for different liposomal encapsulation technologies. Purpura et al. [37] investigated whether a liposomal form of vitamin C (LipoVantage^®^) could enhance absorption compared to placebo and ascorbic acid. Through a randomized, double-blind, placebo-controlled, crossover trial with 27 participants, the researchers measured plasma and leukocyte vitamin C concentrations at 0, 0.5, 1, 1.5, 2, 3, 4, 6, 8, 12, and 24 h after ingestion of a single 500 mg dose of each form. Both standard and liposomal vitamin C significantly increased vitamin C levels compared to placebo (*p*  <  0.001). However, the liposomal form resulted in even higher concentrations in both plasma and leukocytes, indicating improved absorption (Cmax [plasma  + 27%, leukocytes  + 20%, *p*  <  0.001] and AUC0–24 h [plasma  + 21%, leukocytes  + 8%, *p*  <  0.001]) compared to standard ascorbic acid [37].

Wen et al. [12] conducted a crossover trial with 11 participants, who first took vitamin C without liposome, followed by a 14-day washout period, then an intervention with liposomal process A vitamin C (with lecithin) and, after another 14-day washout, liposomal process B vitamin C (with lecithin and high-pressure homogenization; Double Nutri^®^) [12]. Vitamin C levels in plasma were measured at baseline and 0.5, 1, 2, 3, 4, and 8 h post-supplementation. The plasma vitamin C concentration for the liposomal process B vitamin C group was higher (7.26 ± 3.52 μg/mL, *p* < 0.01) compared to the liposomal process A group (6.41 ± 3.80, *p* < 0.05) and the non-liposomal vitamin C group (2.21 ± 4.07, *p* < 0.05).

Gopi and Balakrishnan [36] conducted an open-label, randomized, single-dose, two-treatment, two-sequence, two-period, two-way crossover oral bioavailability study on 24 participants with 1000 mg liposomal vitamin C compared to non-liposomal vitamin C (ascorbic acid) [36]. Orally delivered liposomal vitamin C was 1.8 times more bioavailable, with higher values for Cmax, AUC0–t, and AUC0–1 h compared to non-liposomal vitamin C.

#### 3.4.4. Sustained-Release Vitamin C

Rajat et al. [31] conducted a randomized, placebo-controlled, parallel-design, pharmacokinetic study [31]. Eighteen participants received either 500 mg of sustained-release vitamin C (C-Fence^®^) or placebo. Mean plasma vitamin C concentrations at 12, 16, and 24 h post-dose were, respectively, 0.60, 0.40, and 0.28 μg/mL above baseline values. Both Cmax and AUC24 h were significantly higher than with placebo (*p* < 0.0001).

### 3.5. Summary of Evidence on the Safety and Tolerability of the Interventions

Out of the thirteen analyzed studies, eight (62%) did not report any adverse events, either for the intervention or the comparators [12,27,30,31,33,35,36,37]. One study reported seven adverse events among the 40 studied participants (four with ascorbic acid, two with Calcium ascorbate EC, and one with placebo), but none of the adverse events were related to the investigational products [16].

The study on sustained-release vitamin C (3000 mg daily for 14 days) did not find significant differences in adverse events between the intervention and placebo groups [29]. An upset stomach was reported by 19% of participants taking sustained-release vitamin C in comparison with 15% in the placebo group; diarrhea was reported by 22% vs. 24%; and all other minor complaints by 39% vs. 37%, respectively.

Van Straten and Josling [28] found an overall low incidence of side effects among the 168 participants in their study. Indigestion occurred most frequently in the placebo group, at 10%, compared to 4% among those receiving 1000 mg Calcium ascorbate EC daily over a 60-day period, while the incidence of heartburn was 4% and 7%, respectively.

Gruenwald et al. [32] specifically studied the effects of Calcium ascorbate EC and ascorbic acid (1000 mg/day for 3 days) on gastrointestinal outcomes in healthy adults sensitive to acidic foods. Out of 50 participants, 28 (56%) reported 88 epigastric adverse effects of mild to moderate intensity, of whom 37.5% were taking Calcium ascorbate EC and 62.5% ascorbic acid [32]. There was a statistically significant difference (*p* < 0.05) in the share of participants rating the tolerability of the supplements as “very good” (72% in the Calcium ascorbate EC group vs. 54% in the ascorbic acid group).

Finally, even when using higher dosages and in participants with acid sensitivity (1000 mg daily for day 1–5, 2000 mg daily for day 6–10), no significant changes were observed in the gastrointestinal symptoms of those taking Calcium ascorbate EC, whereas ascorbic acid was associated with increases in abdominal pain (*p* = 0.02), diarrhea (*p* = 0.047), and worsening of the overall Gastrointestinal Symptom Rating Scale score (*p* = 0.019) [34]. Additionally, over the 10-day period, the Role Physical and Bodily Pain scores were significantly improved in the Calcium ascorbate EC group compared to the ascorbic acid group (*p* = 0.004 and *p* = 0.008, respectively).

### 3.6. Quality of the Studies

The findings of the quality assessment conducted using the NICE checklist tool are presented in Table 4 for all the included studies. From the assessment of the methodological quality of thirteen RCTs, ten (77%) showed a low risk of bias [12,16,27,28,29,30,31,32,35,37,38], two (15%) showed a high risk of bias [33,36] and one (8%) showed an unclear risk of bias [34].

## 4. Discussion

This SLR included outcomes from healthy participants from thirteen studies with the mean age across the studies ranging between 24 and 48 years. Among the analyzed forms of vitamin C, more than half of the studies (54%) evaluated Calcium ascorbate EC (Ester-C^®^) as the intervention, while the lipid metabolite form of ascorbic acid was evaluated in only one study. Most studies compared Calcium ascorbate EC with ascorbic acid and placebo, with one study also comparing Calcium ascorbate EC with vitamin C lipid metabolites, ascorbic acid, and calcium ascorbate.

Overall, the quality of the studies was deemed acceptable, with 77% having low risk of bias. None of the studies suggested safety or tolerability concerns for any of the alternative supplemental forms, beyond the most common adverse events already known to be experienced due to intake of high doses of ascorbic acid (gastrointestinal adverse events including nausea, heartburn, epigastric pain, abdominal cramps, and diarrhea) [32], attributable to the acidity and osmotic effects of unabsorbed ascorbic acid passing through the intestine [39].

The assessment of the safety and tolerability profile of the Calcium ascorbate EC (Ester-C^®^) form of vitamin C identified significantly fewer incidents of epigastric adverse events, likely due to its neutral pH as compared to regular ascorbic acid [32]. One study showed that Calcium ascorbate EC was significantly better tolerated in nearly three-quarters of the participants who rated their experience as “very good” as compared with slightly more than half of the participants taking ascorbic acid. Fewer epigastric adverse events were caused by this form relative to ascorbic acid, while preserving the pharmacologic effects is beneficial to those prone to epigastric complaints [32]. Another double-blinded cross-over study by Ye et al. [34] also corroborated these findings, wherein participants taking ascorbic acid faced significant adverse gastrointestinal symptoms, while no change was reported in those taking Calcium ascorbate EC, thus indicating better tolerability with this form of vitamin C [34]. The quality of life was also improved, with significantly superior physical and body pain scores. A post hoc analysis reported that Calcium ascorbate EC induced a favorable oxalate change compared to ascorbic acid [33].

Regarding efficacy, the studied outcomes showed significant variability, preventing an adequate comparison of results. Most studies focused on safety, tolerability, and plasma concentration rather than vitamin C retention in cells and tissues, limiting conclusions about the supplements’ benefits for the immune system. Only four studies (31%) reported leukocyte vitamin C concentration, three (in four articles) for Calcium ascorbate EC [16,27,30,38] and one for liposomal-encapsulated vitamin C [37]. Calcium ascorbate EC appears to promote better vitamin C retention in leukocytes [16,27].

None of the studies reported on vitamin C retention in tissues, which is a significant limitation. While the plasma and serum levels of vitamin C reflect dietary intake, efficacy is better determined by its retention in cells, such as leukocytes, which more accurately reflect tissue vitamin C concentrations [40,41]. Therefore, it is noteworthy that better leukocyte retention of the Calcium ascorbate EC form of vitamin C was reported in some studies [16,27]. Several authors recommend using other blood and tissue markers of vitamin C to predict clinical outcomes, as plasma/serum and red blood cell levels of vitamin C are tightly controlled, and vitamin C levels can differ up to 100-fold between plasma and various tissues [16,27,42,43].

Four included studies reported vitamin C concentration in both plasma and leukocytes for different formulations. Two studies examined 1000 mg doses [16,27], and two examined lower doses (250 and 500 mg) [30,37]. At the 1000 mg dose, the tested vitamin C interventions were significantly different from placebo regarding both plasma and leukocyte vitamin C concentrations [16,27]. Significant differences were found across preparations in leukocyte vitamin C concentrations, favoring Calcium ascorbate EC, but no differences were observed in plasma vitamin C levels [16,27]. Non-linear relations were noted between changes in vitamin C concentration in plasma and leukocytes [16,27]. A significant finding from these studies is that vitamin C metabolites can enhance leukocyte vitamin C retention without altering plasma levels [16,27]. This might be due to a novel mechanism for an enhanced absorption and bioavailability of vitamin C over 24 h, whereby vitamin C enters leukocytes by first converting to dehydroascorbic acid and then back to ascorbic acid upon cell entry [27].

The study by Dickerson et al. further supports these findings, showing that the ingestion of 500 mg Calcium ascorbate EC significantly increased dehydroascorbic acid in plasma, enhanced neutrophil functionality, and promoted an increase in natural killer cells compared to ascorbic acid [30]. The study found no significant differences between Calcium ascorbate EC and ascorbic acid at the 250 mg dose. However, at the 500 mg dose, Calcium ascorbate EC showed greater volume distribution and clearance from blood, suggesting enhanced bioavailability that might relate to the observed immune benefits [30].

Consuming 500 mg of a liposomal-coated source of L-ascorbic acid led to significantly higher Cmax (plasma + 27%, leukocytes + 20%) and 24 h AUC (plasma + 21%, leukocytes + 8%) compared to ascorbic acid, suggesting greater vitamin C absorption into plasma and leukocytes with such formulations [37]. However, this may not necessarily occur for all liposomal forms due to the high variability in terms of liposome size and composition [44].

The variability in the design of the studies included in this systematic review prevents us from properly establishing superiority of one form of vitamin C over others in improving immunity in healthy adults. However, relevant variations were observed in the robustness of the evidence supporting the benefits of each alternative vitamin C form, which are noteworthy.

The studies on sustained-release vitamin C were all against placebo, disabling any conclusion to be made on its benefits over ascorbic acid or other, advanced formulations such as Ester-C^®^, etc. [29,31]. More evidence is needed to establish the potential benefits of this novel formulation on improving immunity.

As for liposomal-encapsulated forms of vitamin C, these encompass different technologies, with studies still at early stages. No adverse events occurred during the included trials [12,36]. In terms of efficacy, orally delivered liposomal vitamin C was found to be 1.8 times more bioavailable than non-liposomal vitamin C (ascorbic acid) [12,36], though this is likely to differ for other liposomal forms due to high variability in the size and composition of the liposomes themselves. While results in terms of bioavailability seem promising, more evidence is needed to enable any conclusion regarding potential benefits on immunity.

Only one trial studied the lipid metabolite form of vitamin C (PureWay-C^®^), comparing it with ascorbic acid, calcium ascorbate, and Calcium ascorbate EC [35]. The study showed that the different forms lead to distinct increases in serum vitamin C level over time, and that vitamin C lipid metabolites led to the highest serum vitamin C levels, with significant increases at 1, 2, 4, and 6 h post-supplementation in comparison with calcium ascorbate. As for Calcium ascorbate EC, significant increases in serum vitamin C levels were observed at 1 and 4 h post-supplementation in comparison with calcium ascorbate. No adverse effects were found based on urine uric acid and oxalate levels. Based on these results, we can only conclude that the type and delivery modality can affect the pharmacokinetic profile, and potentially the functionality, of vitamin C in the body, and that both new forms of vitamin C are probably superior in some ways to calcium ascorbate. The fact that serum and not plasma was used in this study does not enable a direct comparison with results from studies measuring plasma concentration. More studies including vitamin C concentration in cells or tissues are required to conclude on the potential immunity benefits from vitamin C lipid metabolites.

Studies using Calcium ascorbate EC reported favorable effects on leukocyte vitamin C concentration [16,27], showed improved immunity (based on cell functions and numbers) [30], and reported fewer colds and a shorter duration of severe cold symptoms compared to placebo [28]. All studies with Calcium ascorbate EC reported favorable safety and tolerability profile, with most displaying fewer adverse events and better tolerability than comparators, even at higher dosages. Outcomes for Calcium ascorbate EC were more consistent across studies and initial studies suggest a potential superiority to ascorbic acid regarding immunity in healthy adults, measured by the increase in leucocyte vitamin C concentration and immune cell numbers and function. Investigation of the effect of acute doses of vitamin C alone, calcium ascorbate with vitamin C metabolites, and placebo on total plasma and leukocyte vitamin C concentrations 24 h post-dosing supports the understanding that vitamin C supplementation enhances ascorbic acid concentration in plasma and leukocytes, with a greater concentration in leukocytes when receiving vitamin C metabolites compared with ascorbic acid alone or placebo [27]. This hints toward a positive feedback mechanism for vitamin C in response to the exposure of certain cells to metabolites or a sentinel signaling event for tissues requiring more of the nutrient, despite strict control in plasma levels.

While these results look encouraging, further studies are warranted for more conclusive interpretations on the relative impact of different vitamin C formulations on immunity. Future studies would benefit from changes in study design, namely including outcomes more directly related to immune function. Based on the limitations that our SLR encountered resulting from the design of the included studies, we recommend some areas of improvement for future research, summarized in Table 5.

While this SLR focused on healthy adults, there is still scope for improvement in future studies by including larger and more diverse cohorts, including those with immune vulnerabilities such as older people. Additionally, including immune stressors, such as intense exercise, medication, and vaccination, among others, could make results on the role of vitamin C supplements in immune-challenged situations more objective [45,46]. Moreover, additional outcomes, such as circulating cell types, activation of cells, study of the cells ex vivo, etc., should be included [45,46]. At a minimum, trials should compare alternative forms of vitamin C with ascorbic acid, and not just placebo, as this, together with greater homogeneity in study design across trials, would enable both direct and indirect treatment comparisons to be conducted. Ideally, trials should also include other alternative forms of vitamin C as comparators, such as calcium ascorbate and vitamin C lipid metabolites. It is key that researchers standardize a set of markers of immunity [45,46] and inflammation [47]. Finally, studies with longer time periods should be considered to better assess efficacy in the immune system and overall safety. As an example, vitamin C supplementation for more than 6 months can impact respiratory function, which could be measured with surveys and standard measures of immunity and health [48].

## 5. Conclusions

In conclusion, this systematic review highlights the potential advantages of newer alternative forms of vitamin C with increased bioavailability, particularly Calcium ascorbate EC (Ester-C^®^), over traditional ascorbic acid. Calcium ascorbate EC generated the largest and highest quality evidence of all the forms reviewed in this SLR. It demonstrated better tolerability and fewer gastrointestinal side effects, making it a preferable option for individuals prone to epigastric complaints. Additionally, this form showed promising results in increasing leukocyte vitamin C concentrations, which is crucial for immune function, whereas vitamin C lipid-metabolites reported increases in serum concentration. It will be interesting to investigate what additional impact it will have on immune function and inflammation or functional markers. Sustained-release and liposomal-encapsulated vitamin C forms show potential, but more robust evidence is needed to confirm their benefits. The findings suggest that the type and delivery modality of vitamin C can significantly impact its bioavailability and functionality, but further research is required to establish its effects on immune health conclusively.

## Figures and Tables

**Table 1:** Inclusion and exclusion criteria.

Category	Inclusion Criteria	Exclusion Criteria
Population	Healthy adults (≥18 years)	Animal or in vitro study, pediatric population, chronic conditions. Chronic smokers. Obese population (BMI > 30 kg/m^2^).
Interventions/comparators	Oral forms of the following: Liposomal-encapsulated ascorbic acid Liposomal-encapsulated lipid metabolite ascorbic acid Calcium ascorbate Slow-release ascorbic acid Lipid metabolite ascorbic acid	Any form/route not mentioned in inclusion criteria. Interventions with multivitamins as they do not enable us to isolate the effect of Vitamin C
Outcomes	Bioavailability, absorption, plasma or serum vitamin C concentrations, retention in white blood cells, impact on immunity or infection, tolerability	Only reports other outcomes
Study design	RCTs, non-RCTs, single-arm studies, and observational studies	Reviews, letters, comments, case reports, case series and editorials
Language of publication	English	Any other language
Country where study was conducted	Any	No exclusion criteria applied
Time of publication	Publication since 2000	Publication prior to 2000

BMI: body mass index; RCT: randomized clinical trial.

**Table 4:** Quality assessment of the included studies.

Intervention	Publication	Sample Size	Randomization	Double Blinding	Cross-Over	Overall Risk of Bias
Calcium ascorbate EC (Ester C^®^)	Dickerson et al., 2024 [30]	93	Yes	Yes	Yes	Low risk
	Mitmesser et al., 2016 * [16]	40	Yes	Yes	Yes	Low risk
	Mitmesser et al., 2014 * [38]	36	Yes	Yes	Yes	Low risk
	Ye et al., 2015 [34]	50	Yes	Yes	Yes	Unclear risk
	Van Straten and Josling 2002 [28]	168	Yes	Yes	No	Low risk
	Gruenwald et al., 2006 [32]	50	Yes	Yes	Yes	Low risk
	Moyad et al., 2008 [27]	15	Yes	Yes	Yes	Low risk
	Moyad et al., 2009 [33]	34	Yes	Not clear	Yes	High risk
Vitamin C lipid metabolites (PureWay C^®^)	Pancorbo et al., 2008 [35]	40	Yes	Yes	No	Low risk
Liposomal-encapsulated vitamin C	Purpura et al., 2024 [37]	27	Yes	Yes	Yes	Low risk
	Wen et al., 2022 [12]	11	Yes	Yes	Yes	Low risk
	Gopi and Balakrishnan 2021 [36]	24	Yes	No	Yes	High risk
Sustained-release vitamin C	Rajat et al., 2022 [31]	18	Yes	Yes	No	Low risk
	Brody et al., 2002 [29]	120 **	Yes	Yes	No	Low risk

* Same study. ** There were no drop-outs from the study but 12 of the 120 subjects (6 from each group) were excluded from analysis.

**Table 5:** Methodological recommendations for future studies.

Improvement Areas	Recommendation
Population	Have larger population size
	Include more diverse populations
	Report outcomes by subpopulation
Intervention/Comparator	Introduce immune stressors
	Ensure comparison at least with ascorbic acid
	Favor having other alternative forms of vitamin C as comparators
Outcomes	Include a standardized set of outcomes more directly related to immune function. These immunological parameters could be, for example, circulating cell types, activation of cells, study the cells ex vivo, immune response to vaccination, etc.
	Standardize the use of surveys and standard measures of immunity and health
	Work toward more standard markers of inflammation and circulating markers
Study design	Increase study duration to better assess efficacy, ideally with at least 6 months of follow-up
	Assess dietary intake of vitamin C before, during, and after the study period

## Data Availability

The original data that support the findings presented in this study are included in the article/Supplementary Materials. Further inquiries can be directed to the corresponding author.

## Supplementary Materials

The following supporting information can be downloaded at: https://www.mdpi.com/article/10.3390/nu17020279/s1, Table S1: Search strategy for Embase and Medline (Search period from 2000–30th Sept 2024. Table S2. Search Strategy for Embase 1974 to 2024 October 28 (Search period: 01 October 2024–28 October 2024). Table S3. Search Strategy for Ovid Medline ALL 1946 to 28 October 2024 (Search period: 01 October 2024–28 October 2024).
