# Supplementary material for: Enhanced Vitamin C Delivery: A Systematic Literature Review Assessing the Efficacy and Safety of Alternative Supplement Forms in Healthy Adults
Source: Nutrients. 2025 Jan 14;17(2):279. doi: 10.3390/nu17020279 (PMC11767823; doi:10.3390/nu17020279)
Supplement: Supplementary file 1 [file nutrients-17-00279-s001.zip › nutrients-3416636-supplementary.pdf]

Supplementary Materials

Enhanced Vitamin C Delivery: a Systematic Literature Review assessing the Efficacy and Safety of Alternative Supplement Forms in Healthy Adults

Philip C. Calder [1,2,*], Richard B. Kreider [3] and Diane L. McKay [4]

Table S1. Search strategy for Embase and Medline (Search period from 2000-30th Sept 2024.

Sno	Searches	Results
1	("vitamin c" or ascorbic acid or Ester C or "Ester-C" or PureWay C or Liposomal Vitamin C or C Fence).ti,ab.	93,939
2	(bioavailab* or plasma concentration* or plasma level* or absorb* or tolerability or immunity or immune or Leukocyte* or liposom* or metabolite* or threonate or slow release or delay* release or sustained-release or retention or gastrointestinal upset or gastro intestinal upset or gastric distention or gastrointestinal outcome* or gastrointestinal discomfort or pharmacokinetics or white blood cells or WBC or encapsulat* or common cold or flu).ti,ab.	4,380,283
3	exp randomized controlled trial/ or randomized controlled trials as topic/ or exp randomization/ or exp clinical trial/ or double blind.ti,ab. or single blind.ti,ab. or (cross-over or crossover).ti,ab. or randomization/ or control group/ or (clin$ adj trial$).ti,ab. or randomi?ed controlled trial$.ti,ab. or RCT.ti,ab. or ((singl$ or doubl$ or trebl$ or tripl$) adj (blind$ or mask$)).ti,ab. or placebo$.ti,ab. or (random$ adj allocat$).ti,ab. or open label.ti,ab. or (phase adj (III or "3") adj (study or studies or trial*)).ti,ab. or ((equivalence or superiority or non-inferiority or noninferiority) adj (study or studies or trial*)).ti,ab. or randomized controlled trial.pt. or (nonrandom* or non random* or non-random* or quasi-random* or quasirandom*).ti,ab.	4,304,205
4	exp observational study/ or exp case control study/ or exp cohort studies/ or exp cross sectional studies/ or exp prospective study/ or exp retrospective study/ or case control.ab,ti. or cohort?.ab,ti. or cross sectional.ab,ti. or ((follow up or longitudinal or retrospective$ or prospective$ or observational or single arm) adj stud*).ti,ab.	8,357,618
5	3 or 4	11,560,589
6	1 and 2 and 5	3,035
7	limit 6 to english language	2,938
8	7 not (exp Neoplasms/ or exp Musculoskeletal Diseases/ or exp Nervous System Diseases/ or exp Eye Diseases/ or exp Cardiovascular Diseases/ or exp Endocrine System Diseases/ or exp Urogenital Diseases/ or exp Surgery/ or exp dermatology/ or exp case report/ or (cancer* or surgery or "in vitro" or tumor* or tumor* or lymphoma* or sarcoma* or carcinoma* or infant* or child* or diabet* or anemia or anemia or skin or skincare or topical or cream or colonoscop* or fruit or juice or dermatology or cardiovascular or cardiac or case series or case report or systematic review or systematic literature review or meta analysis or COVID or protocol or kiwifruit* or mice or bowel preparation or school or blueberr* or periodont* or toddler* or obese or obesity or overweight or pleurotus or tea or adolescent* or pregnan* or maternal or maternity or fish oil or student* or breastfeed* or breast feed* or pediatric* or pediatric* or HIV or preschool* or hearing loss or quercetin or vagina* or tinnitus or stent or broccoli or player* or athlet* or gestation* or fibrosis or transplant* or leprosy or cardio* or mutation* or sunscreen or menopaus* or postmenopaus* or cocoa or crohn* or spectr* or cholesterol*).ti. or (letter or comment or editorial or note or commentary or case report or review).pt.)	899
9	limit 8 to yr="2000 -Current"	804
10	remove duplicates from 9	562

Table S2. Search Strategy for Embase 1974 to 2024 October 28 (Search period: 01 October 2024 - 28 October 2024).

Sno	Searches	Results
1	("vitamin c" or ascorbic acid or Ester C or "Ester-C" or PureWay C or Liposomal Vitamin C or C Fence).ti,ab.	70215
2	(bioavailab* or plasma concentration* or plasma level* or absorb* or tolerability or immunity or immune or Leukocyte* or liposom* or metabolite* or threonate or slow release or delay* release or sustained-release or retention or gastrointestinal upset or gastro intestinal upset or gastric distention or gastrointestinal outcome* or gastrointestinal discomfort or pharmacokinetics or white blood cells or WBC or encapsulat* or common cold or flu).ti,ab.	3183494
3	exp randomized controlled trial/ or randomized controlled trials as topic/ or exp randomization/ or exp clinical trial/ or double blind.ti,ab. or single blind.ti,ab. or (cross-over or crossover).ti,ab. or randomization/ or control group/ or (clin$ adj trial$).ti,ab. or randomi?ed controlled trial$.ti,ab. or RCT.ti,ab. or ((singl$ or doubl$ or trebl$ or tripl$) adj (blind$ or mask$)).ti,ab. or placebo$.ti,ab. or (random$ adj allocat$).ti,ab. or open label.ti,ab. or (phase adj (III or "3") adj (study or studies or trial*)).ti,ab. or ((equivalence or superiority or non-inferiority or noninferiority) adj (study or studies or trial*)).ti,ab. or randomized controlled trial.pt. or (nonrandom* or non random* or non-random* or quasi-random* or quasirandom*).ti,ab.	3087261
4	exp observational study/ or exp case control study/ or exp cohort studies/ or exp cross sectional studies/ or exp prospective study/ or exp retrospective study/ or case control.ab,ti. or cohort?.ab,ti. or cross sectional.ab,ti. or ((follow up or longitudinal or retrospective$ or prospective$ or observational or single arm) adj stud*).ti,ab.	5148566
5	3 or 4	7497985
6	1 and 2 and 5	1926
7	limit 6 to english language	1860
8	7 not (exp Neoplasms/ or exp Musculoskeletal Diseases/ or exp Nervous System Diseases/ or exp Eye Diseases/ or exp Cardiovascular Diseases/ or exp Endocrine System Diseases/ or exp Urogenital Diseases/ or exp Surgery/ or exp dermatology/ or exp case report/ or (cancer* or surgery or "in vitro" or tumor* or tumor* or lymphoma* or sarcoma* or carcinoma* or infant* or child* or diabet* or anemia or anemia or skin or skincare or topical or cream or colonoscop* or fruit or juice or dermatology or cardiovascular or cardiac or case series or case report or systematic review or systematic literature review or meta analysis or COVID or protocol or kiwifruit* or mice or bowel preparation or school or blueberr* or periodont* or toddler* or obese or obesity or overweight or pleurotus or tea or adolescent* or pregnan* or maternal or maternity or fish oil or student* or breastfeed* or breast feed* or pediatric* or pediatric* or HIV or preschool* or hearing loss or quercetin or vagina* or tinnitus or stent or broccoli or player* or athlet* or gestation* or fibrosis or transplant* or leprosy or cardio* or mutation* or sunscreen or menopaus* or postmenopaus* or cocoa or crohn* or spectr* or cholesterol*).ti. or (letter or comment or editorial or note or commentary or case report or review).pt.)	518
9	limit 8 to dd=20241001-20241028	1
10	limit 8 to rd=20241001-20241028	2
11	9 or 10	3

Table S3. Search Strategy for Ovid Medline ALL 1946 to October 28, 2024 (Search period: 01 October 2024 - 28 October 2024).

Sno	Searches	Results
1	("vitamin c" or ascorbic acid or Ester C or "Ester-C" or PureWay C or Liposomal Vitamin C or C Fence).ti,ab.	61578
2	(bioavailab* or plasma concentration* or plasma level* or absorb* or tolerability or immunity or immune or Leukocyte* or liposom* or metabolite* or threonate or slow release or delay* release or sustained-release or retention or gastrointestinal upset or gastro intestinal upset or gastric distention or gastrointestinal outcome* or gastrointestinal discomfort or pharmacokinetics or white blood cells or WBC or encapsulat* or common cold or flu).ti,ab.	2441702
3	exp randomized controlled trial/ or randomized controlled trials as topic/ or exp randomization/ or exp clinical trial/ or double blind.ti,ab. or single blind.ti,ab. or (cross-over or crossover).ti,ab. or randomization/ or control group/ or (clin$ adj trial$).ti,ab. or randomi?ed controlled trial$.ti,ab. or RCT.ti,ab. or ((singl$ or doubl$ or trebl$ or tripl$) adj (blind$ or mask$)).ti,ab. or placebo$.ti,ab. or (random$ adj allocat$).ti,ab. or open label.ti,ab. or (phase adj (III or "3") adj (study or studies or trial*)).ti,ab. or ((equivalence or superiority or non-inferiority or noninferiority) adj (study or studies or trial*)).ti,ab. or randomized controlled trial.pt. or (nonrandom* or non random* or non-random* or quasi-random* or quasirandom*).ti,ab.	1903951
4	exp observational study/ or exp case control study/ or exp cohort studies/ or exp cross sectional studies/ or exp prospective study/ or exp retrospective study/ or case control.ab,ti. or cohort?.ab,ti. or cross sectional.ab,ti. or ((follow up or longitudinal or retrospective$ or prospective$ or observational or single arm) adj stud*).ti,ab.	4224252
5	3 or 4	5682044
6	1 and 2 and 5	1595
7	limit 6 to english language	1552
8	7 not (exp Neoplasms/ or exp Musculoskeletal Diseases/ or exp Nervous System Diseases/ or exp Eye Diseases/ or exp Cardiovascular Diseases/ or exp Endocrine System Diseases/ or exp Urogenital Diseases/ or exp Surgery/ or exp dermatology/ or exp case report/ or (cancer* or surgery or "in vitro" or tumor* or tumor* or lymphoma* or sarcoma* or carcinoma* or infant* or child* or diabet* or anemia or anemia or skin or skincare or topical or cream or colonoscop* or fruit or juice or dermatology or cardiovascular or cardiac or case series or case report or systematic review or systematic literature review or meta analysis or COVID or protocol or kiwifruit* or mice or bowel preparation or school or blueberr* or periodont* or toddler* or obese or obesity or overweight or pleurotus or tea or adolescent* or pregnan* or maternal or maternity or fish oil or student* or breastfeed* or breast feed* or pediatric* or pediatric* or HIV or preschool* or hearing loss or quercetin or vagina* or tinnitus or stent or broccoli or player* or athlet* or gestation* or fibrosis or transplant* or leprosy or cardio* or mutation* or sunscreen or menopaus* or postmenopaus* or cocoa or crohn* or spectr* or cholesterol*).ti. or (letter or comment or editorial or note or commentary or case report or review).pt.)	590
9	limit 8 to dd=20241001-20241028	2
10	limit 8 to rd=20241001-20241028	6
11	9 or 10	6
